# Engineering and Exploring Hydrolytic Degradation in 3D-Printed Liquid Crystalline Elastomers

**DOI:** 10.1021/acs.biomac.5c02638

**Published:** 2026-04-03

**Authors:** Lorin C. Danielsen, Jason A. Burdick, Timothy J. White

**Affiliations:** † Department of Chemical and Biological Engineering, 1877 University of Colorado Boulder, Boulder, Colorado 80303, United States; ‡ BioFrontiers Institute, University of Colorado Boulder, Boulder, Colorado 80303, United States; § Materials Science and Engineering Program, University of Colorado Boulder, Boulder, Colorado 80303, United States

## Abstract

Liquid crystalline elastomers (LCEs) are being increasingly explored as biomaterials; however, many LCE properties were not designed with biomedical use in mind. Here, we examine LCE hydrolytic degradation, including investigating approaches to accelerate degradation and whether thermal and mechanical properties change with degradation. Among 3D-printable LCE chemistries, we find that networks formed via thiol-Michael addition followed by thiol–ene photo-cross-linking degrade most rapidly. The integration of hydrophilic chain extenders (e.g., PEG) accelerates LCE degradation and demonstrates their potential tunability for various applications. We monitor representative LCEs throughout degradation and show that as samples undergo heterogeneous surface erosion, nematic-to-isotropic transition temperatures increase, while actuation potential, alignment, and mechanical anisotropy remain stable until failure. ^1^H NMR, SAXS, and DSC studies reveal that thermal changes arise from retained degradation products enriched in liquid crystal mesogens, which increase mesogenic interactions per unit volume and require greater thermal energy to disrupt the nematic state.

## Graphical abstract

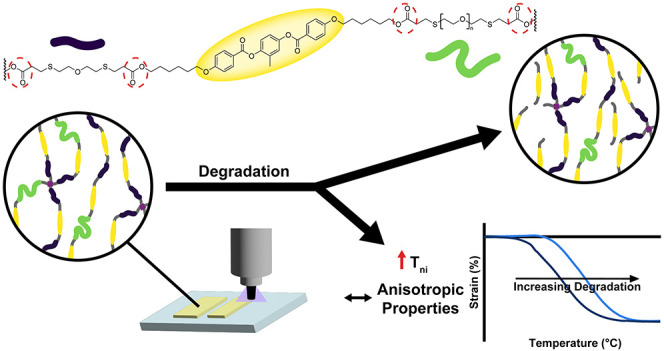

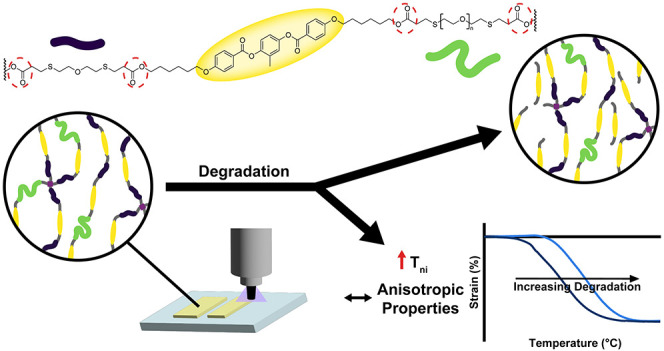

## 1 Introduction

Liquid crystalline elastomers (LCEs) are soft polymer networks that combine the mechanics of elastomers with the molecular order of liquid crystals.^1^ This coupling allows changes in liquid crystal order to produce controlled changes in shape and mechanical response. The direction and magnitude of these responses can be set by programming alignment during synthesis or subsequent processing. LCEs also retain anisotropy in mechanical properties, with moduli as much as an order of magnitude higher parallel to the alignment direction than orthogonal to it. Accordingly, LCEs have been studied as artificial muscles,^2,3^ soft robotic actuators,^4–6^ and damping materials for mechanical energy management.^7,8^ In these applications, maintaining long-term stability is typically prioritized, since degradation could diminish performance.

More recently, interest has expanded toward biological systems, where cells and tissues interact directly with LCEs. One theme across these studies is the use of liquid crystal network anisotropy to guide cell growth and organization.^9–14^ Surface-aligned liquid crystal polymers have been shown to direct myoblast alignment,^9,10^ and photopatterned, swollen LCEs have been used to guide fibroblast organization.^11^ Beyond cell–level interactions, LCE properties such as temperature responsivity and shape-changing behavior have been leveraged in biomedical devices.^15–17^ These include dynamic LCE-based urologic support devices^16^ and subcutaneous LCE implants capable of reversible photothermal reconfiguration.^17^ Together, these studies illustrate the promise of using LCEs as functional biomaterials.

These investigations of LCEs fit within a broader context of anisotropic and temperature-responsive biomaterials. Anisotropic systems, like oriented fibrous scaffolds,^18,19^ micropatterned substrates,^20^ and aligned porous architectures^21,22^ have all been used to guide cell alignment, migration, and tissue organization. In these systems, the anisotropy is typically fixed after fabrication and is maintained as long as the material remains structurally stable. Temperature-responsive biomaterials like lower critical solution temperature (LCST) polymers^23–25^ and shape memory polymers^26,27^ have been employed for applications in drug delivery, tissue engineering scaffolds, and smart cell culture substrates. For these materials, response is coupled to a material phase change, making them dependent on chemical content, but unreliant on molecular or macroscopic orientation. LCEs are unique in that their anisotropy and stimuli-responsivity are associated with a temperature dependent phase change in addition to molecular order within the network that can be programmed during processing. This introduces the possibility that anisotropy may evolve if the underlying chemistry or mesogen content changes. As a result, understanding how degradation affects alignment, thermal transitions, and mechanical anisotropy in LCEs becomes particularly important, especially as these materials are increasingly considered for biological use in biomedical devices,^28^ tissue engineering scaffolds,^29^ and drug delivery platforms.^30^

Many common LCE chemistries contain hydrolytically labile bonds such as esters, suggesting that hydrolysis could meaningfully influence LCE performance over time.^1,31,32^ However, LCE degradation has rarely been examined beyond simple mass loss measurements.^33–35^ One exception is a study that investigated LCE degradation under acidic conditions that simulate an oxidizing environment in the body.^36^ This environment was shown to oxidize electron-rich bonds and promote bond hydrolysis, which complicates analysis of hydrolytic degradation.^17^ Additionally, the study mainly focused on how oxidation induces programmed shape changes, leaving many open questions regarding the mechanisms and consequences of hydrolytic degradation on LCEs.

Here, we investigated hydrolytic degradation in LCEs by (i) evaluating three 3D-printable LCE chemistries, (ii) introducing strategies to accelerate the rate of LCE degradation, and (iii) examining how and why LCE properties change with hydrolytic degradation. Due to this process proceeding over long time scales, we used an accelerated hydrolytic degradation environment of sodium hydroxide (NaOH) to address these questions, which is a common method for accelerating and understanding the degradation of hydrophobic biomaterials in vitro.^37^ This approach enabled the rapid comparison of hydrolytic degradation across formulations, while the inclusion of controls at neutral pH helps to understand LCE degradation and performance under more representative conditions. Ultimately, this work expands our understanding of LCE hydrolytic degradation beyond mass loss, allowing an understanding of mechanistic changes in thermal and mechanical properties with degradation that can help inform the future of LCE biomaterial design and provide general insights into LCE hydrolytic stability.

## 2 Experimental Section (Materials and Methods)

### 2.1 Materials

All chemicals were used as received and purchased from various sources: sodium hydroxide (NaOH, Fisher Scientific), 1,4-*Bis*[4-(6-acryloyloxyhexyloxy)­benzoyloxy]-2-methylbenzene (C6M (also referred to as RM82), Wilshire Technologies), *bis*(2-mercaptoethyl) ether (BMEE, TCI America), poly­(ethylene glycol) dithiol MW600 (PEG, Creative PEGWorks), *N*,*N*′-dimethylhexane-1,6-diamine (DMHDAM, Combi-Blocks), glyoxal bis­(diallyl acetal) (GBDA, Sigma-Aldrich), dipropylamine (DPA, Sigma-Aldrich), 1,6-hexanedithiol (HDT, Sigma-Aldrich), 4-methoxyphenol (MEHQ, Sigma-Aldrich), Irgacure 369 (I-369, photoinitiator, IGM Resins), acetone (Sigma-Aldrich), phosphate-buffered saline (PBS, Dulbecco’s).

### 2.2 Oligomerization Reactions

Oligomers were prepared by first melt-mixing solid reactants in a vial using a heat gun followed by pipetting liquid reactants directly into the melt. After vortexing, the material was placed on a hot plate at 85 °C for 18 h for the aza-Michael reaction and at 65 °C for 3 h for the thiol-Michael reactions. A table comparing the formulations for each of these chemistries can be found in Table S1.

### 2.3 Bulk LCE Synthesis

To create bulk polydomain samples, oligomers were spread between RainX (a commercial polymer solution that adheres to glass and creates a hydrophobic coating which aids in the easy removal of polymerized LCEs) coated glass slides with 25 μm plastic spacers. The slides were created by dabbing on liquid RainX with a Kimwipe, allowing the solution to dry for about 15 s, and then buffing the slides with a dry Kimwipe to create an even coating. Samples were then irradiated with 125 mW/cm^2^ of 365 nm light for 10 min. In the study to compare the three different LCE chemistries, samples were cross-linked 10 °C below their oligomer *T*_ni_ upon cooling (Figure S1). The remaining samples in this work were cross-linked at room temperature as the *T*_ni_ could not be reached without cooling below room temperature when high concentrations of PEG were introduced. After removal from slides, the samples were cut into 6 mm diameter circles using a biopsy punch.

### 2.4 3D-Printing of LCEs

3D-printing was conducted on a Hyrel3D System 30 M printer using a KR2 printhead and custom low-volume HTK print cartridges. Oligomers were heated to 65 °C, loaded into the printhead, and heated with a heat gun for 30 min to remove any bubbles. The ink was then allowed to cool to room temperature before a 400 μm nozzle was screwed onto the tip and the printhead was loaded into the printer. To achieve uniform prints, the printhead was heated to 37 °C and the print bed was heated to 30 °C. Rectangles were then printed onto poly­(vinyl alcohol) (PVA) coated slides at a print speed of 6 mm/s with a layer height of 150 μm. During printing, the rectangles were irradiated with 0.8 mW/cm^2^ of 365 nm light to lock in the alignment imparted by rheological forces. Directly after printing, the rectangles were postcured with 125 mW/cm^2^ of 365 nm light for 10 min to finish cross-linking. 21 mm × 2.5 mm rectangles were printed for the majority of samples and 21 mm × 20 mm rectangles were printed and cut perpendicular to the path of extrusion (and resulting molecular alignment) into approximately 20 mm × 2.5 mm strips for use in perpendicular tensile testing. Polydomain samples were not cured with UV light during printing but were instead heated after printing until they reached the isotropic phase and lost alignment, then cooled to room temperature and cross-linked with 125 mW/cm^2^ of 365 nm light for 10 min.

### 2.5 Degradation of LCEs

Degradation studies were conducted in PBS, as well as 10, 1, and 0.1 M NaOH solutions. After weighing the initial dry samples, they were submerged in these solutions and placed in an incubator at 37 °C. The solution surrounding the samples was changed every 7 days. At each time point, samples were measured using calipers, rinsed with deionized water, dried in an oven at 37 °C, and then weighed. Dry mass remaining was determined using the following equation where *m*_d_ is the mass of the dried degradation sample and *m*_0_ is the initial dry mass of the sample

$$\mathrm{Dry}\;\mathrm{mass}\;\mathrm{remaining}\;\left(\%\right)=100\%\times \frac{m_{d}}{m_{0}}$$

Water content relative to dry degraded mass was determined using the following equation where *m*_h_ is the mass of the hydrated degradation sample and *m*_d_ is the mass of the dried degradation sample

$$\mathrm{Water}\;\mathrm{content}\;\left(\%\right)=100\%\times \left(m_{h}-m_{d}\right)/\left(m_{d}\right)$$

Length change relative to initial length was determined using the following equation where *l*_h_ is the length of the hydrated degradation sample and *l*_0_ is the initial dry length of the sample

$$\mathrm{Length}\;\mathrm{change}\;\left(\%\right)=100\%\times \left(l_{h}-l_{0}\right)/\left(l_{0}\right)$$

Since POM imaging, tensile testing, and X-ray scattering experiments required hydrated samples, at each time point some samples were just rinsed with deionized water and then stored in PBS until analysis. Day 0 hydrated samples for tensile testing were produced by soaking samples in PBS overnight at 37 °C.

### 2.6 Differential Scanning Calorimetry

Approximately 5 mg of material was loaded into a hermetically sealed aluminum pan for each sample. DSC experiments were performed on a TA Instruments Discovery DSC 2500. Samples were heated to 180 °C, cooled to −50 °C, and heated to 180 °C once again at a rate of 5 °C/min. The reported data are from the second heating step.

### 2.7 Polarized Optical Microscopy

A Nikon Eclipse Ci-Pol with a 50× objective was used to capture images of unstrained, hydrated samples at 0° and 45°. The presence of birefringence indicates molecular alignment.

### 2.8 Scanning Electron Microscopy

Dried samples were imaged on a Hitachi TM-4000PlusE-2 SEM at 100× magnification using a secondary electron detector. The samples were held under medium vacuum with an accelerating voltage of 15 kV.

### 2.9 Actuation Testing via Dynamic Mechanical Analysis

Dried samples approximately 2.5 mm in width were loaded into clamps approximately 10 mm apart on a TA Instruments DMA 850. Samples were exposed to a preload force of 0.005 N, cooled to −30 °C, equilibrated for 5 min, and then heated to 150 °C at a rate of 5 °C/min. Strain values were normalized to a sample’s strain at 0 °C.

### 2.10 Tensile Testing

Hydrated samples 0.10 mm in thickness were cut to approximately 1.25 mm in width to ensure tests could be run to completion were loaded into clamps approximately 5 mm apart on a TA Instruments RSA-G2. Tests were run at 5% strain/min with a maximum preload force of 0.0125 N. The Young’s modulus was calculated as the slope of the first 0.1–2% of the initial linear elastic region of the stress–strain curves.

### 2.11 X-ray Scattering

Wide angle X-ray scattering (WAXS) and small-angle X-ray scattering (SAXS) experiments were conducted on a Xenocs Xeuss 3.0 on both dry and hydrated samples. WAXS and SAXS experiments were conducted with exposure times of 1 and 5 min, respectively. *D*-spacing was calculated based on *q* values using Bragg’s law

$$d=\frac{2\pi }{q}$$

Hermans orientation parameter (*S*) was calculated from WAXS data using the 1D data reduction module in the Xenocs software and a custom MATLAB script using the following equations where *χ* is azimuthal angle and *I­(χ)* is intensity as a function of azimuthal angle

$$\left\langle \cos^{2}\chi \right\rangle =\frac{\int_{0}^{\pi /2}I\left(\chi \right)\;\cos^{2}\chi \;\sin\chi \;d\chi }{\int_{0}^{\pi /2}I\left(\chi \right)\;\sin\chi \;d\chi }$$

$$S=\frac{3\left\langle \cos^{2}\chi \right\rangle -1}{2}$$

### 2.12 Removal and Characterization of Degradation Products

Samples were soaked in acetone overnight, washed in deionized water, dried in a 37 °C oven overnight, and weighed. Acetone was evaporated under a chemical hood to obtain the retained degradation products. Mass removed from the degraded samples with acetone relative to the dried degradation sample mass was calculated using the following equation where *m*_r_ is the mass removed from the sample with acetone and *m*_d_ is the mass of the dried degradation sample

$$\mathrm{Mass}\;\mathrm{removed}\;\left(\%\right)=100\%\times \left(m_{r}\right)/\left(m_{d}\right)$$

Some of these products were then resuspended in chloroform at 1 mg/mL for GPC (Tosoh EcoSEC HLC-8230) against polystyrene standards, with 280 nm UV absorption detection. Other products were resuspended in deuterated chloroform for ^1^H NMR analysis (400 MHz Bruker spectrometer).

Day 0 samples with C6M swollen into the network were prepared by dissolving C6M in acetone and then soaking samples in the mixture overnight. The samples were then washed in deionized water, placed in a 37 °C oven to dry overnight, and weighed.

### 2.13 Statistical Analysis

Data are reported as mean ± SD with *n* ≥ 3. GraphPad Prims 10 (GraphPad Inc., USA) was used to perform all statistics. A two-way analysis of variances (ANOVA) with Tukey’s multiple comparison test was used to compare more than two experimental groups with two independent variables.

## 3 Results and Discussion

### 3.1 Degradation of 3D-Printable LCE Systems

Aligned LCEs prepared by rheological alignment via direct ink write (DIW) 3D-printing are commonly synthesized using a two-step reaction process. The first step involves an addition reaction to form long oligomer chains, followed by a second step consisting of a free radical cross-linking reaction to convert these oligomers into a polymer network.^1,31,38^ Three common chemistries are compatible with DIW including aza-Michael, thiol-Michael, and thiol-Michael/thiol–ene (Figure 1a).^1,31^ A common theme in all three reactions is the use of Michael addition to oligomerize commercially available diacrylate liquid crystal monomers, or mesogens. For aza-Michael systems, the oligomerization step involves the reaction of difunctional amine monomers with diacrylate mesogens. An excess of acrylate functional groups yields acrylate capped oligomers that can be processed and subsequently cross-linked into a polymer network via free-radical acrylate photopolymerization. Thiol-Michael systems are similar, except oligomerization is achieved through a base-catalyzed thiol-Michael addition between difunctional thiol monomers and diacrylate mesogens. In this system, excess acrylate functional groups again result in acrylate capped oligomers that can be subsequently cross-linked. Thiol-Michael/thiol–ene systems also use the base-catalyzed thiol-Michael addition for oligomerization but are prepared with excess thiol functional groups to produce thiol terminated oligomers. These oligomers can then be cross-linked with an alkene terminated multifunctional cross-linking molecule via free-radical thiol–ene photopolymerization.

**Figure 1:**
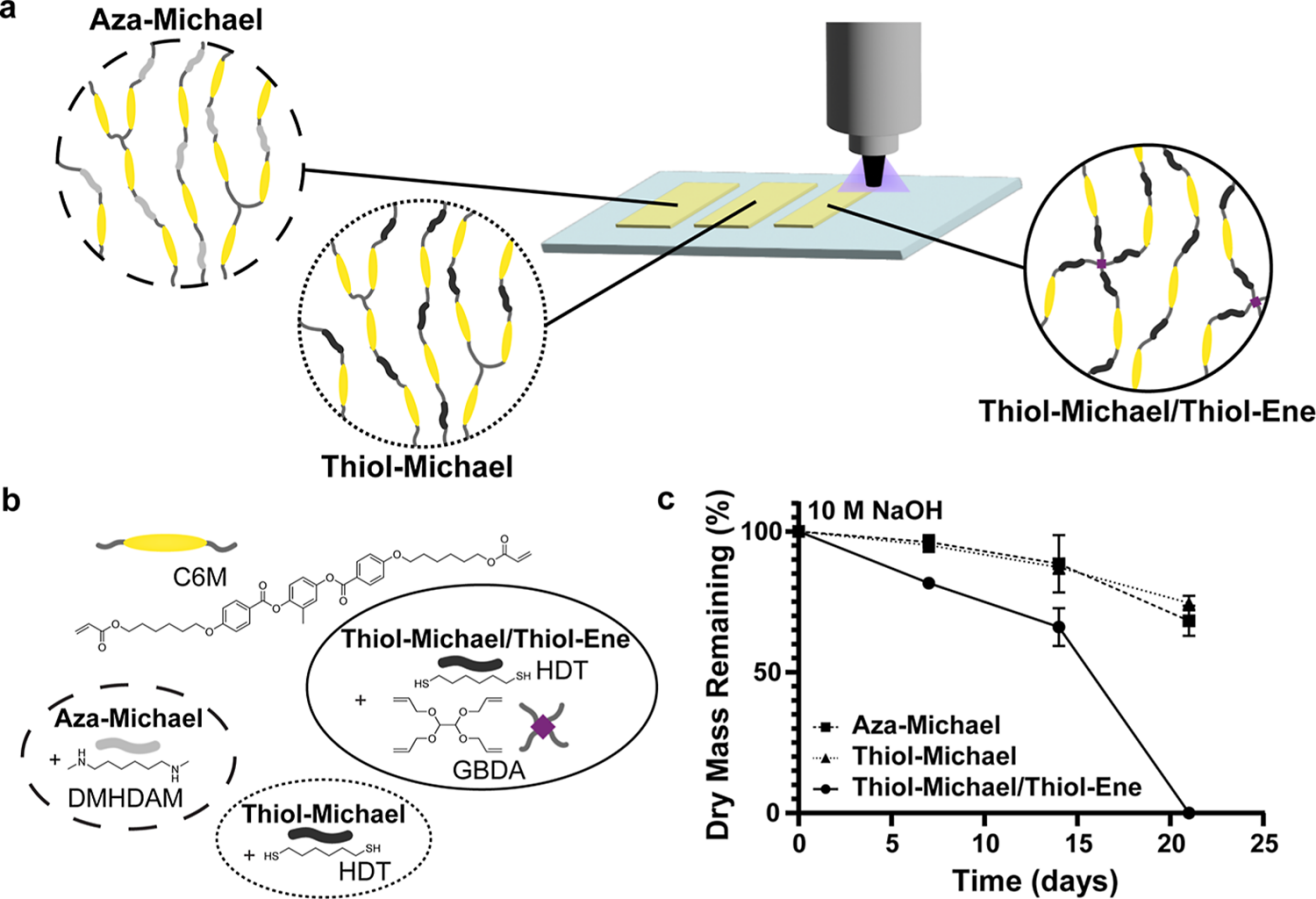
Comparison of three 3D-printable LCE chemistries. (a) Network structures for the different chemistries, including aza-Michael, thiol-Michael, and thiol-Michael/thiol–ene systems; (b) monomers used for each of these systems that incorporate the C6M mesogen and allow for controlled polymerizations; (c) mass loss for bulk samples of each formulation during degradation in 10 M NaOH. Data are reported as mean ± SD; *n* = 3.

To compare the hydrolytic degradability of these three systems, formulations were designed with specific monomer ratios and structures to result in similar theoretical molecular weights between cross-links and all incorporating the same mesogen (C6M) (Figure 1b and Table S1). Polydomain LCE samples were prepared and degraded in 10 M NaOH at 37 °C. This concentration was selected after observing negligible mass loss in 1 M NaOH over 35 days (Figure S2). The high NaOH concentration needed to initiate appreciable degradation in these LCEs suggests that these chemistries in their basic forms are very hydrolytically stable and may be poor options for use as degradable biomaterials without further engineering. However, these studies are necessary to provide a baseline for subsequent materials development.

Among the three chemistries, the thiol-Michael/thiol–ene system degraded the fastest, with samples losing roughly 18.4% of their mass by day 7, roughly 33.9% by day 14, and finally losing overall mechanical integrity and fragmenting apart after 21 days (Figure 1c). The aza-Michael and thiol-Michael systems showcased markedly less degradation and also behaved similarly, with no statistically significant differences between the two at any time points. They showed around 3.8% and 4.9%, 11.5% and 12.8%, and 31.7% and 25.5% mass loss at these time points, respectively. The similarities between the degradation profiles of the two acrylate cross-linked systems indicate that the choice of cross-linking reaction has a greater effect on degradation rate than the oligomerization reaction. This may be attributed to the thiol–ene cross-links incorporating additional ethers into the network, but it is also potentially due to a difference in conversion during cross-linking. In previous studies, two-step acrylate cross-linked LCE systems showed nearly 100% acrylate functional group conversion using real time Fourier transform infrared spectroscopy.^39^ Conversion in two-step thiol–ene cross-linked LCE systems has not been studied as thoroughly, but other LCEs prepared with thiol–ene photopolymerization showed much lower conversions,^40^ as well as reduced moduli compared to other LCE chemistries,^41^ which is often an indicator of lower cross-linking.^42^ These factors likely increase the accessibility of esters to water, which increases the rate of hydrolytic cleavage. We additionally note that the aza-Michael system lacks functional groups susceptible to oxidation, meaning that various biological interactions (e.g., immune response) would likely not expedite degradation.^43^ Given these results we selected the thiol-Michael/thiol–ene system for the remaining work in this study.

### 3.2 Increasing the Degradation Rate of Thiol-Michael/Thiol–Ene LCEs

Different biological applications often demand different rates of biomaterial degradation.^32^ For example, drug delivery systems may require degradation over days, months, or years, while tissue engineering scaffolds should degrade at rates similar to the rate of tissue formation. In contrast, biomedical devices often call for a complete lack of degradation in the body. Therefore, it is of interest to be able to tune biomaterial degradation for specific applications. Since the LCEs explored demonstrate extremely slow degradation, we were motivated to investigate methods to accelerate their degradation.

Given the high hydrophobicity of the mesogen structures, we hypothesized that increasing the hydrophilic content in the LCE network would increase their rate of hydrolytic degradation. To test this, the chain extender molecule HDT, which mainly consists of a long aliphatic chain and was used above, was exchanged for BMEE and PEG dithiol (MW 600) at various ratios (Figure 2a,b). BMEE and PEG dithiol contain either a single or a chain of ether groups, respectively, which increase network polarity and improve water penetration and therefore could alter network hydrolysis. Polydomain samples were prepared from these adjusted compositions and placed in 0.1 M NaOH at 37 °C for degradation.

**Figure 2:**
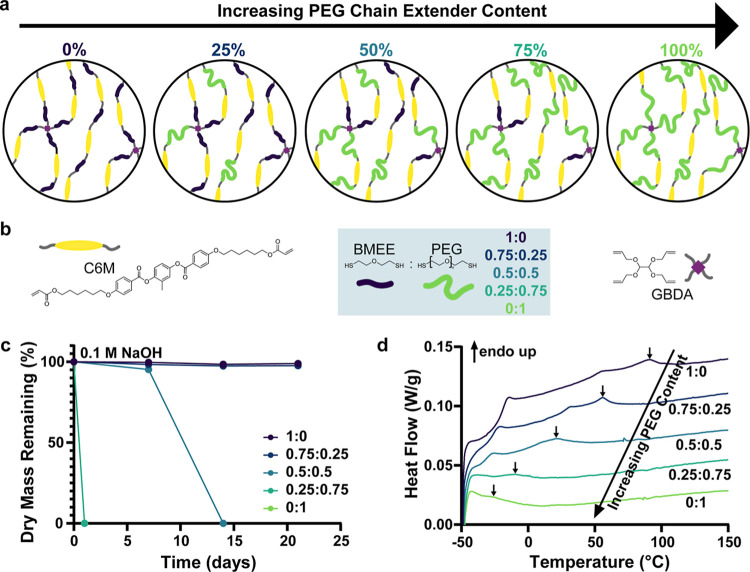
Increasing the rate of hydrolytic degradation for thiol-Michael/thiol–ene networks. (a) Network structures formed by altering the molar ratio of chain extenders *bis*(2-mercaptoethyl) ether (BMEE) to PEG-dithiol (PEG) from 1:0, 0:75:0.25, 0.5:0.5, 0.25:075, to 0:1 where network hydrophilicity increases with more PEG incorporation; (b) monomers used in network formation, including the C6M mesogen, BMEE and PEG chain extenders (five distinct chain extender ratios noted), and glyoxal *bis*(diallyl acetal) (GBDA) cross-linker; (c) mass loss for bulk samples of each of the five formulations during degradation in 0.1 M NaOH (note that the 0:1 formulation is hidden beneath the 0.25:0.75 formulation and error bars are hidden by the circular symbols for all data points); (d) DSC traces for each formulation (arrows indicate the location of each *T*_ni_). Data are reported as mean ± SD; *n* = 3.

As predicted, increasing the PEG content resulted in an increased rate of degradation and an increase in network hydrophilicity (Figures 2c and S3). The formulations with only PEG or 75% PEG used as the chain extender degraded and fragmented apart within a day, the formulation with 50% PEG showed 4.8% mass loss after 7 days and lost mechanical integrity in 14 days, and the formulations with 25% or 0% PEG lost little mass within 3 weeks. It should be noted that this increase in degradation with the incorporation of PEG is also coupled with a decrease in liquid crystal content, due to the increased molecular weight of the PEG molecule relative to the alternate chain extender BMEE. This is particularly important as previous studies have demonstrated that reducing liquid crystal content in LCE networks diminishes their stimuli-response and achievable degree of molecular orientation.^44–46^ This trend is reflected in the DSC traces for these formulations, where increased PEG content is associated with a less pronounced nematic to isotropic transition peak and a shift to lower temperatures, ranging from 90 °C to well below 0 °C (Figure 2d). While these results demonstrate that hydrophilicity can be tuned to increase degradation rates, the inherent hydrophobicity of the mesogens may restrict water accessibility to the ester groups and result in a functional trade-off.

### 3.3 Effect of Hydrolytic Degradation on LCE Properties

We selected the 0.75:0.25 BMEE:PEG material for further investigation, due to its retention of a high concentration of mesogens, which results in a *T*_ni_ above room temperature and close to body temperature. Based on the degradation profile shown above, this formulation would likely be more applicable for longer-term degradation applications. DIW 3D-printing was used to fabricate single-layer, rectangular samples, which were then degraded in 1 M NaOH at 37 °C to overcome the hydrolytic stability of the formulation that was demonstrated in 0.1 M NaOH (Figure 3a). The samples showed gradual mass loss over a 10 day period accompanied by increasing water uptake, a common occurrence during hydrolytic degradation (Figure 3b).^32^ After 8 days and roughly 58.1% mass loss, the samples began to lose mechanical integrity and fractured when manipulated with tweezers. By day 10, they fragmented into small pieces without agitation, preventing collection and measurement, which may alter accurate quantification of mass loss (Figure S4). The mass loss profile is also notably different from the profile for the same formulation in Figure 2, likely due to the higher concentration of NaOH used and the nonuniform surface features created during DIW 3D printing, which can increase the effective surface area and change degradation solution interactions with the samples when compared to those polymerized in bulk. For comparison, PBS samples showed significantly less mass loss at only about 1.8% after 150 days.

**Figure 3:**
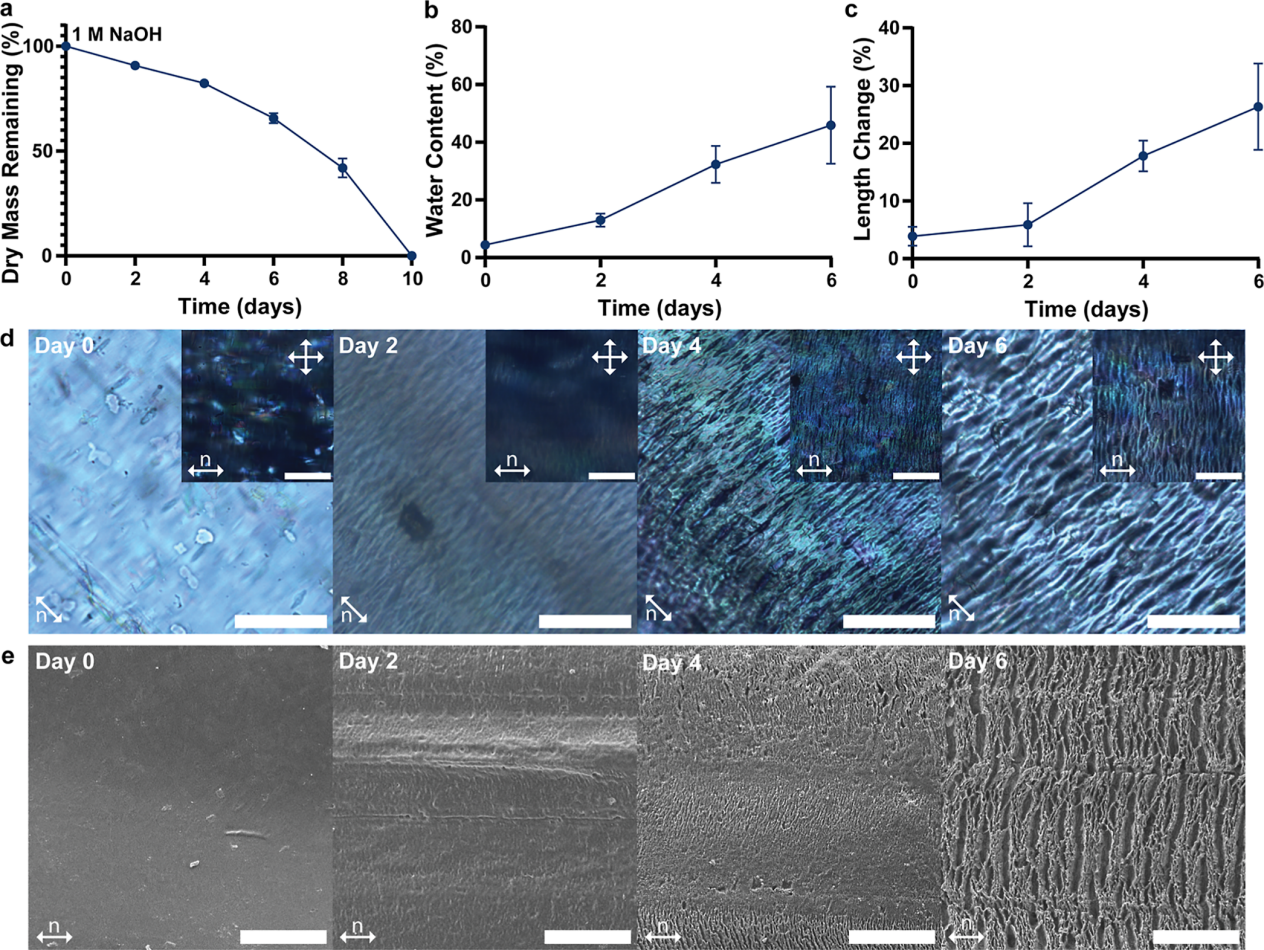
Degradation of 0.75:0.25 BMEE:PEG 3D-printed samples in 1 M NaOH. (a) Mass loss; (b) water content relative to degraded dry mass; (c) length change relative to initial sample lengths; (d) polarized optical microscopy (POM) images of unstrained, hydrated samples at 0° (smaller image) and 45° (larger image) offset from cross polarizers, scale bar = 50 μm; (e) SEM images of dried samples, scale bar = 250 μm. Data are reported as mean ± SD; *n* = 3.

The samples also demonstrated anisotropic lengthening in the direction of mesogen alignment during degradation, increasing in length by more than 26.4% by day 6 (Figures 3c and S5). While shape changes in LCEs are often associated with a liquid crystal to isotropic transition, this is unlikely in our samples due to the direction of sample lengthening. An expansion perpendicular to alignment indicates mesogens moving out of an aligned liquid crystal state and into an isotropic state.^1^ POM images also showed birefringence that remained brighter at a 45° offset relative to the axis of polarized light, indicating the maintenance of a monodomain, aligned liquid crystal phase (Figure 3d). Together, these observations suggest that the lengthening arises from degradation-related structural changes rather than a loss of liquid crystal order.

We propose that this lengthening is due to anisotropic surface cracking, as evidenced by the POM and SEM images, as well as the relief of surface stresses during degradation (Figure 3d,e). Degradation solution uptake by hydrophilic domains increases internal stresses, which may be dissipated through the initiation of cracks along the weaker mechanical axis, perpendicular to the axis of polymer chain alignment, like those that grow in number and size from days 2 to 6.^47,48^ This cracking may also then relieve residual surface stresses from material processing (e.g., photopolymerization, 3D printing), which results in material relaxation and macroscopic lengthening.^49,50^ The uptake of water into these surface pores, may exert additional expanding surface stresses on the samples. Importantly, polydomain samples exhibited less sample lengthening and showed rounder and more isotropic surface erosion features, confirming that mesogen alignment drives this anisotropic behavior during degradation (Figure S6). Even though the mechanism behind this lengthening cannot be entirely elucidated without further experimentation, the persistence of these dimensional changes indicates a maintenance of mechanical anisotropy and alignment throughout degradation and highlights a potential design consideration for LCE biomaterials.

DSC revealed that *T*_ni_ increases with degradation, a trend also observed in the 150 day PBS controls (Figures 4a and S7a). The peaks around 20 °C in each time point suggest the presence of an additional mesophase transition, although the temperature of this transition was not affected by degradation. For the nematic peak, there was a large initial increase in *T*_ni_ between days 0 and 2 of about 15.1 °C and more modest increases between days 2, 4, and 6 at around 3.8 and 5.6 °C, respectively (values in Table S2). From day 0 to 4, the transition peaks appear consistent in shape and area, but starting at day 6 the peak area begins to increase. Peak area is proportional to the transition’s enthalpy, which is essentially the amount of energy required to disrupt the current material state.^51^ Thus, this trend suggests that mesogen interactions may change at later time points. By day 8, the transition peaks changed shape, size, and location more drastically, which is likely related to the concurrent loss of mechanical integrity.

**Figure 4:**
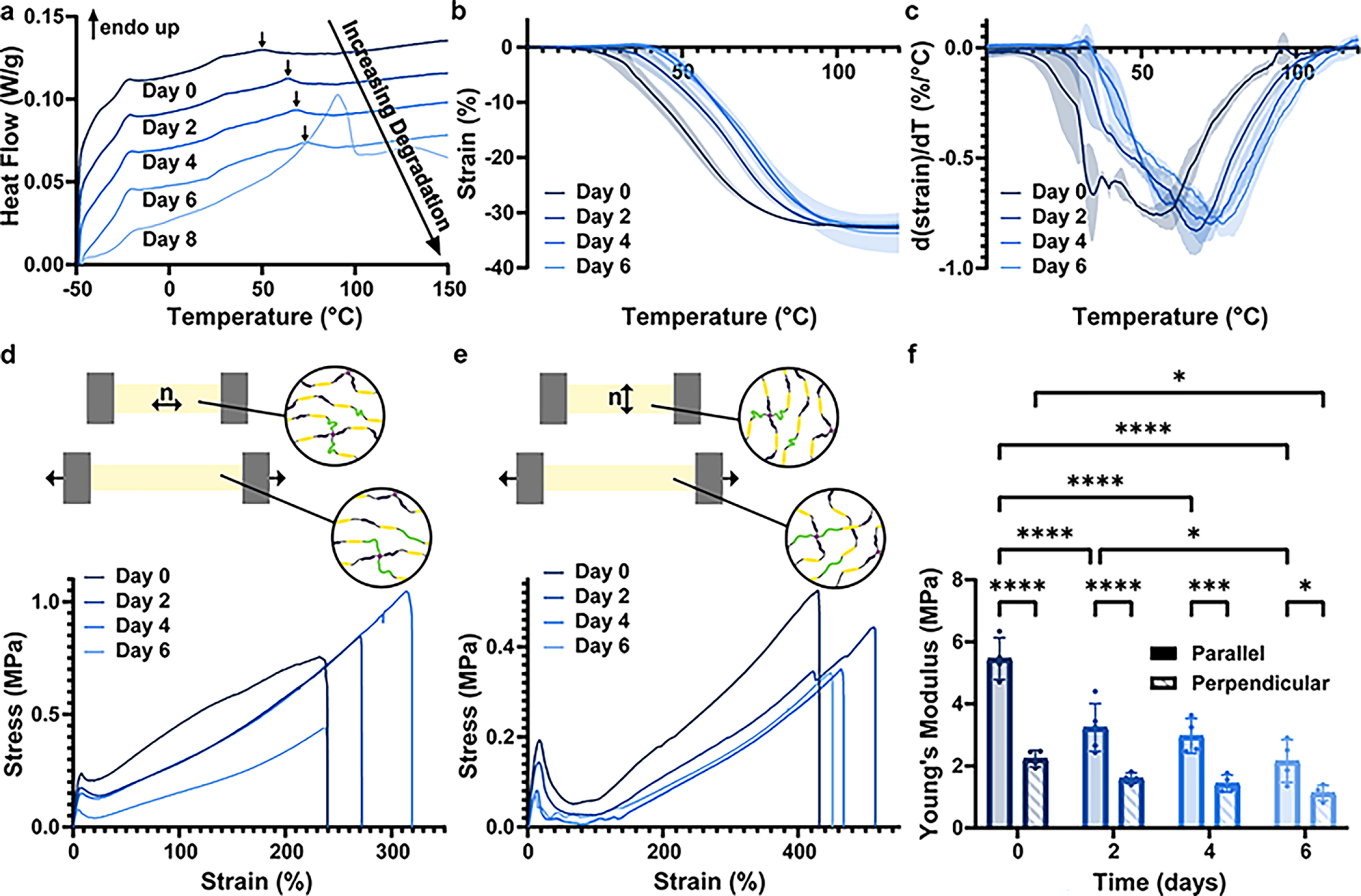
Thermal and mechanical characterization of 0.75:0.25 BMEE:PEG degraded (1 M NaOH) 3D-printed samples. (a) DSC traces (arrows indicate the location of each *T*_ni_, besides day 8 due to the change in transition behavior); (b) actuation strain; (c) first derivative of actuation strain curves; (d) representative tensile tests parallel to mesogen alignment; (e) representative tensile tests perpendicular to mesogen alignment; (f) Young’s moduli values in parallel (solid) and perpendicular (striped) bars. Data are reported as mean ± SD; *n* = 3 (a–c); *n* ≥ 4 (f); *p < 0.05; ***p < 0.001; ****p < 0.0001.

Isostress tests, or actuation tests, corroborated the findings surrounding *T*_ni_. The temperature corresponding to the maximum rate of actuation, another metric of *T*_ni_, increased with degradation for both the accelerated and PBS control samples (Figures 4b,c and S7b). The results also showed that an actuation potential of −32.6% strain and maximum rate of actuation of around −0.8%/°C were maintained throughout degradation, implying that underlying molecular alignment and network structure remain largely intact prior to loss of mechanical integrity.^46^

Tensile testing revealed that sample hydration has a plasticizing effect on LCEs, evidenced by the peak at the end of the initial strain regime associated with sample yielding and unrecoverable deformation.^32,52^ The dry, undegraded day 0 samples did not exhibit this yielding peak when tested parallel to mesogen alignment and showed a more weakly pronounced peak perpendicular to mesogen alignment (Figure S8). The representative tensile curves parallel and perpendicular to the director showed that samples maintain similar tensile behavior throughout degradation (Figure 4d,e). Together with the actuation data, these results indicate that the network response remains directionally consistent even as hydrolytic degradation progresses.

Young’s moduli values decreased from 5.5 to 2.2 MPa parallel to the direction of mesogen alignment and from 2.2 to 1.1 MPa perpendicular to the direction of mesogen alignment over the first 6 days of degradation (Figure 4f). Importantly, the ratio of parallel Young’s modulus to perpendicular Young’s modulus remained constant around a value of 2 across these time points. These results indicate material softening while mechanical anisotropy and consequently mesogen alignment are maintained. However, since the sample thickness did not change drastically with degradation, these findings likely suggest that cracking and subsequent heterogeneous erosion at the sample surface preserves the apparent cross-sectional area, while the portion of the sample core that remains intact decreases over time. The work conducted by this intact region is likely the same as an entire undegraded sample if normalized to its smaller cross-sectional area, highlighting the role of a degraded outer shell in the apparent material softening. This interpretation is consistent with the anisotropic cracking behavior observed in Figure 3.

Collectively, these functional results indicate that LCE degradation is more surface rather than bulk dominated. Accelerated degradation in NaOH is often hypothesized to favor this effect due to the degradation process becoming more dominated by the rate of bond hydrolysis rather than by water penetration into the material.^53,54^ However, our results here are also supported by PBS controls and prior observations in vivo and are likely encouraged by the high hydrophobicity of these materials.^17^ Importantly, this mode of degradation results in the preservation of the majority of LCE functional properties throughout degradation, besides an increase in *T*_ni_. This change in thermal properties motivated us to further explore what is happening to these networks during degradation on a molecular level.

### 3.4 Network Changes during Degradation at the Molecular Length Scale

To better understand the molecular structure of these degrading LCEs, we conducted WAXS experiments to investigate lateral mesogen spacing and to evaluate mesogen alignment (Figure 5a).^55,56^ The consistent peak *q* value over the first 6 days indicates that lateral distance between mesogens was maintained (Figure 5b). Orientation parameters for both the dry and hydrated samples, 0.47 and 0.30, respectively, also remained consistent through day 6 and in PBS control samples, indicating that alignment is not significantly affected by degradation (Figures 5c and S13b). Alignment only began to decline at day 8, coinciding with the loss of sample mechanical integrity.

**Figure 5:**
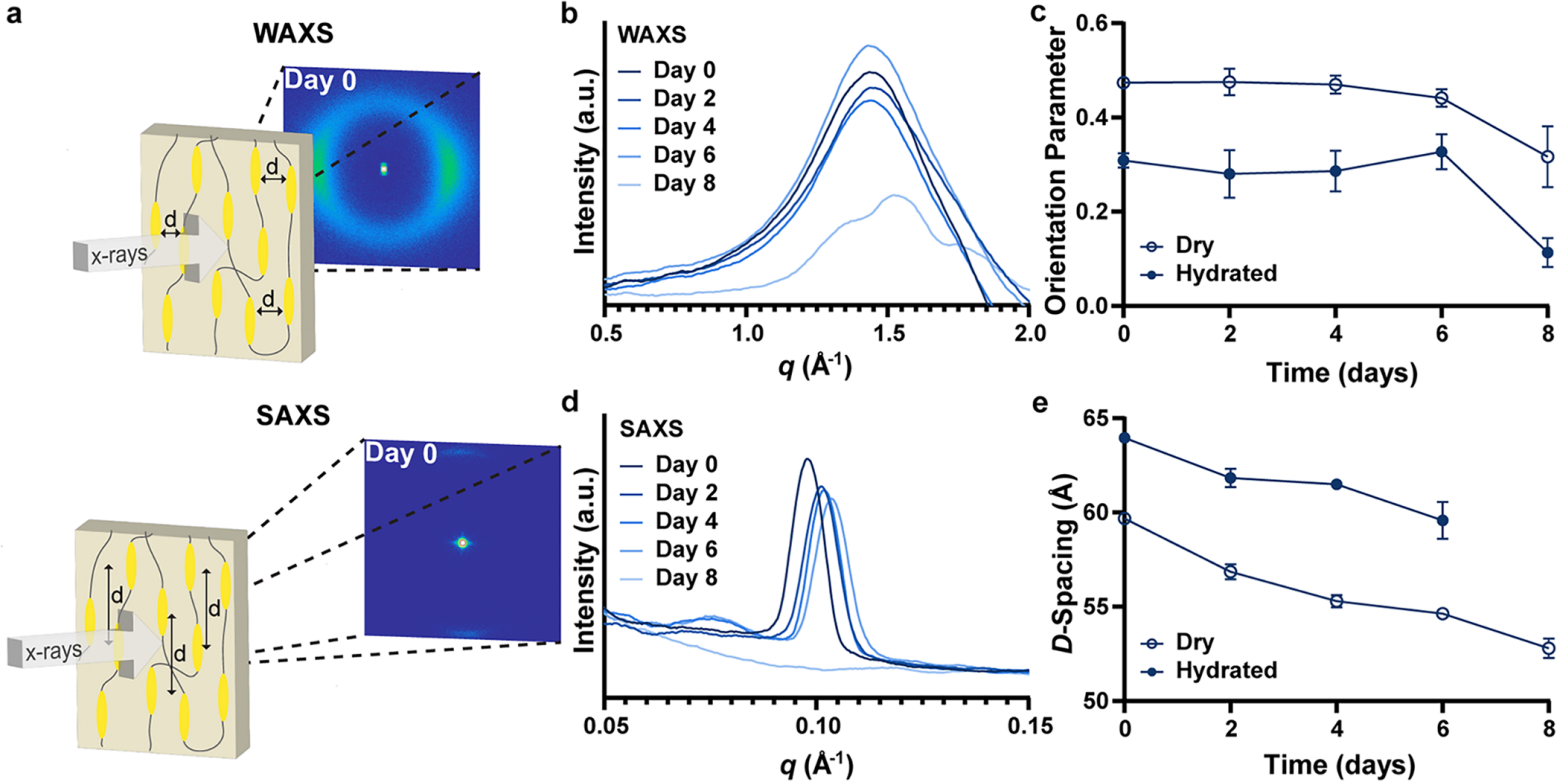
WAXS and SAXS characterization of 0.75:0.25 BMEE:PEG degraded (1 M NaOH) 3D-printed samples. (a) Diagrams of WAXS and SAXS experiments on LCEs where *d* denotes *d*-spacing, a distance in angstroms; (b) WAXS traces; (c) orientation parameter, with solid circles representing hydrated and empty circles representing dry samples; (d) SAXS traces; (e) *d*-spacing corresponding to SAXS peaks, with solid circles representing hydrated and empty circles representing dry samples. Data are reported as mean ± SD; *n* = 3.

SAXS experiments were also performed to assess changes in longitudinal mesogen spacing with degradation (Figure 5a).^55^ In both accelerated and PBS degradation, the main SAXS peak shifted to higher *q* values, indicating that mesogens packed together end-to-end more closely with degradation (Figures 5d,e and S13c). Mesogens moved from an average spacing of 64.0 to 59.6 Å for hydrated samples from days 0 to 6 and from 59.7 to 52.8 Å for dry samples from days 0 to 8. This difference in spacing suggests that the intact LCE network uptakes some amount of water, but its consistency also reveals that the water uptake during degradation reported in Figure 3 is likely to be due to an increase in surface porosity rather than absorption into the LCE network. This trend supports the *T*_ni_ increases observed in Figure 4 as it indicates that degradation enriches local mesogen density, which would increase thermal energy requirements to disrupt the nematic state.

### 3.5 Removal and Characterization of Degradation Products

Since functional testing points to little change in the internal cross-linked network structure with degradation, changes in *T*_ni_ and SAXS *d*-spacing are likely due to the presence of noncovalently attached degradation products being retained near or in the core of the samples. We propose that degradation primarily occurs at the mesogen terminal esters dividing the mesogen and amorphous portions of the network due to the hydrophobic shielding of the central core esters. This results in the production of hydrophilic fragments that can dissolve into the degradation solution and mesogen-rich fragments that may be retained in the sample due to their insolubility and desirable interactions with surrounding mesogen cores. The accumulation of these fragments causes a relative increase in mesogen content within samples. This process parallels how crystallinity increases in semicrystalline polymers with hydrolytic degradation. Through a process called chemi-crystallization, degraded fragments capable of crystallization that were previously entangled or trapped in amorphous regions can crystallize into new domains or with nearby pre-existing crystalline domains, increasing the relative crystalline content of the network.^57^

To investigate this, we washed nondegraded and degraded samples in acetone and saw a 9.5%, 39.3%, and 50.5% increase in the mass of noncovalently attached fragments at each time point relative to day 0, respectively (Figure 6a). In other words, the gel fraction decreases with degradation. In the PBS control this change was not significant, likely due to the small amount of degradation and the elution of a portion of the original sol fraction during degradation (Figure S14).

**Figure 6:**
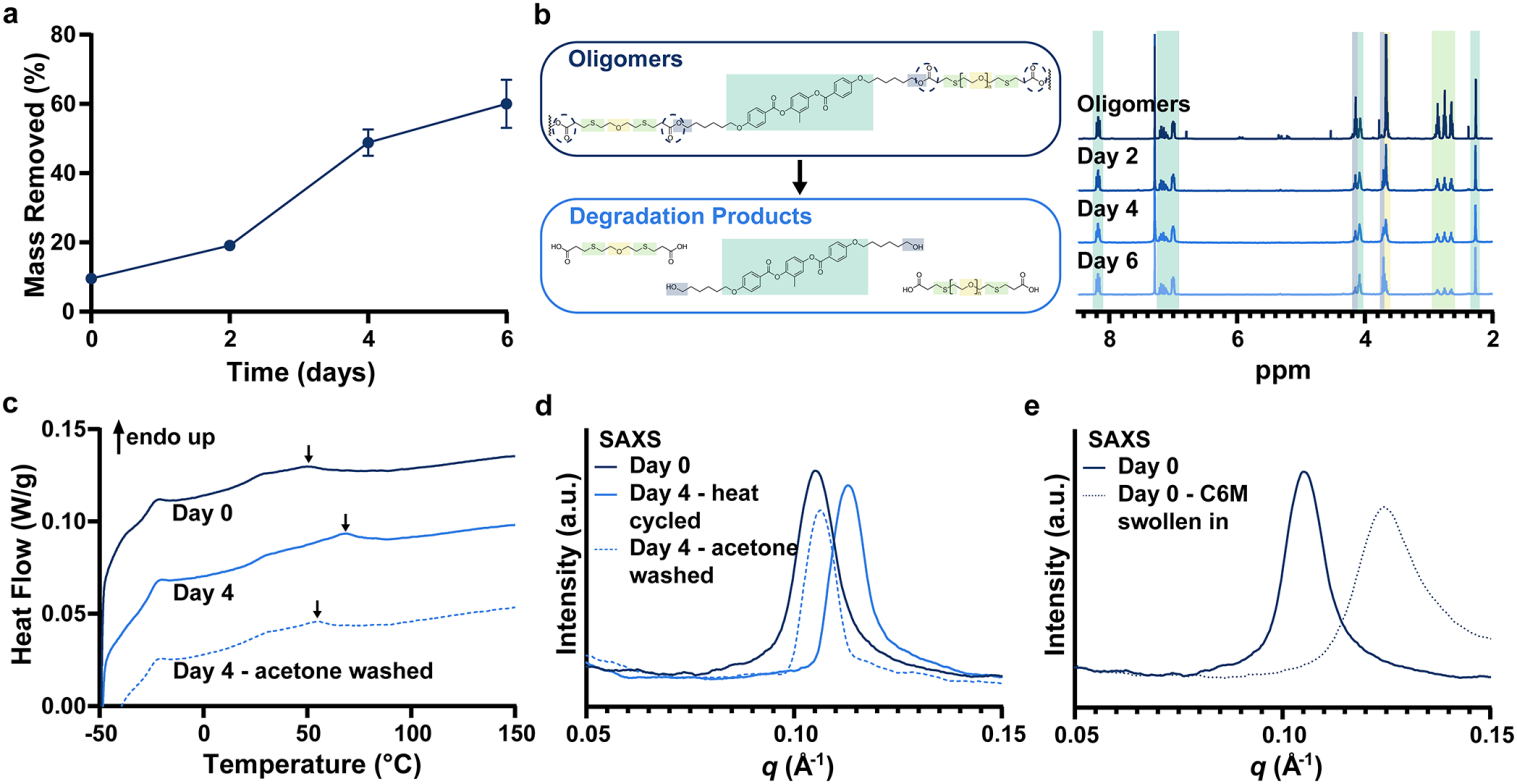
Removal and characterization of degradation products for 0.75:0.25 BMEE:PEG degraded (1 M NaOH) 3D-printed samples. (a) Percent dry mass removed from dry samples with acetone; (b) chemical structures for oligomers and potential degradation products and ^1^H NMR traces for oligomers and degradation products removed from dry samples; (c) DSC changes due to degradation product removal (arrows indicate the location of each *T*_ni_); (d) SAXS changes due to degradation product removal; (e) SAXS traces for day 0 and day 0 with C6M swollen in (dotted) samples. Data are reported as mean ± SD; *n* = 3.

We then characterized the chemical content of the products removed from the degraded samples using ^1^H NMR at each time point and compared it to the composition of the original oligomer chains (Figure 6b). We observed a consistent decrease in peak areas associated with ether and thiol functional groups, with roughly 87.8% and 74.7% reductions by day 6, respectively, and the growth of a peak related to the hydroxyl groups terminating degraded mesogens. The accompanying maintenance of peaks related to the mesogen core indicates a significantly higher ratio of mesogen content to non-mesogen content in the retained degradation products as degradation proceeds.

We additionally characterized the molecular weights of these products using GPC (Figure S15). The traces showed that increased degradation resulted in a decline in the amount and size of large oligomer fragments and an increase in products smaller than single mesogens, which indicates that degradation was perhaps also occurring at the central core esters. However, these degraded core fragments are not evident in the ^1^H NMR data as the original peak locations for the aromatic protons in the mesogen core are retained and their conversion to carboxylic acid, hydroxyl, or acetal groups would likely result in peak shifts. This suggests that the degraded mesogen core fragments evident in the GPC make up a small portion of the retained degradation products. These fragments are perhaps more readily soluble in NaOH or their cleavage happens at a significantly slower rate than the other esters in the system. However, based on the trends in the GPC data, we predict that the prevalence of mesogen core degradation could increase as degradation proceeds even further.

The similarity of the primary degradation products with intact mesogen cores to C6M and previously investigated liquid crystal mesogens in literature, suggests they can likely display a nematic phase and interact with other mesogen cores through pi–pi stacking.^58,59^ By day 6, more than 50% of the remaining material is made up of these retained fragments, yet orientation parameter and birefringence are both consistently maintained, meaning these fragments must be aligning with the mesogen cores in the underlying LCE.

After removing these fragments, we also conducted DSC experiments on day 0, day 4, and day 4 samples washed in acetone. We observed a dramatic shift back toward day 0 thermal properties, with *T*_ni_ reverting from 18 °C higher than day 0 at day 4 to just 4.3 °C after washing, indicating that these fragments are interacting with and strengthening the existing liquid crystal phase (Figure 6c). These DSC results also reinforce the idea that the internal core of the samples remain largely unaffected by degradation and degradation is surface dominated. The slight increase from day 0 properties retained after washing is likely due to the failure to remove all degraded fragments, the presence of mesogen terminated dangling ends, and the preferential excision of PEG over BMEE chain extenders due to their higher concentration of hydrophilic ether groups. The lack of change in the earlier mesophase transition also indicates that only the transition to the isotropic phase appears to be affected by the presence of these fragments. At day 8 when the material no longer contains the guiding template of an aligned LCE network, we see a drop in orientation parameter as shown in Figure 5 and the DSC trace begins to be reminiscent of a DSC trace for unreacted C6M as shown in Figure 4, which has a sharp crystalline melting peak around 85 °C.^60^ The increase in DSC peak area that we observed starting at day 6 may be consistent with these fragments, now comprising the majority of the material, beginning to significantly crystallize with one another and perhaps undergoing chemi-crystallization similar to semicrystalline polymers during hydrolytic degradation. The increasing presence of degraded core fragments and the decline in longer oligomer fragments being retained in the material, as visible in the GPC data, may also contribute to this crystallization behavior. These results indicate that the material trends toward being entirely composed of noncovalently bound mesogens, both whole and beginning to fragment further.

We then assessed if these fragments were responsible for the changes in SAXS *d*-spacing. Similar to the DSC results, the removal of the degradation products shifted the SAXS peak location closer to that of the day 0 peak (Figure 6d). Washing LCEs in organic solvents has been shown to induce a liquid crystal to isotropic transition, however a day 4 sample retained its peak position after heating and cooling through this same transition.^61^ Thus, a liquid crystal to isotropic transition is not responsible for this change in spacing. Finally, we substantiated this idea by swelling in additional C6M mesogen into a nondegraded sample. These additional mesogens caused a large leftward shift of the peak, similar to the presence of degradation products (Figure 6e). Together, these experiments confirm the presence of degraded, mesogen-rich fragments that are retained after degradation and directly link their accumulation to the observed increase in *T*_ni_ and longitudinal packing.

### 3.6 Limitations

Accelerated degradation of biomaterials in an alkaline environment is a common approach for understanding how hydrophobic, or slow degrading, polyester-based biomaterials may change due to hydrolytic degradation.^37,54,62,63^ We want to recognize that this environment lacks many biological factors that can influence degradation in vivo and leaves lingering questions about the effects of enzymes and oxidizing species on degradation, as well as the cytocompatibility of LCEs and their degradation products when released under physiological conditions.^32,37^ These factors are all significantly influenced by the location of biomaterial implantation in the body and are variable from organism to organism, which makes it difficult to model in vitro. However, based on the work of others, while we would reasonably expect enzymatic degradation to result in the cleavage of hydrolyzable groups (e.g., esters), the material’s hydrophobicity and cross-linking would likely restrict enzyme activity throughout the material.^37,62^ The oxidative degradation of LCEs, which can occur in high acidity environments or due to immune response, has also been addressed previously. For example, Javed et al. explored LCE degradation due to oxidation of thioethers under accelerated oxidative degradation, or high acidity, conditions.^36^ Also, Skillin et al. used a very similar LCE formulation to ours and showed that these materials elicited an immune response comparable to medical grade silicone and postulated that reactive oxidizing species effectively accelerated the rate of ester cleavage by increasing local hydrophilicity at the sample surface.^17^ Lastly, due to the very long length scale of degradation shown in our controls in PBS, there is minimal release of degradation products that would influence cell behavior to understand cytotoxicity in vitro. Ultimately, this work proposes how nematic transition temperature, birefringence, SAXS spacing, and mechanical anisotropy change during degradation and future work will be needed to investigate cytotoxicity and biocompatibility within a specific application.

## 4 Conclusion

Degradation is an important parameter in the design of materials, including those for biological applications. We identified formulations that permitted degradation vis hydrolysis, by investigating different LCE oligomerization and cross-linking reactions, as well as through the introduction of hydrophilic chain extenders. While mesogen hydrophobicity limits LCE hydrolytic degradability, it simultaneously promotes a more surface dominated degradation mechanism that helps preserve LCE functional properties throughout degradation. This likely means that during short in vitro experiments the material will remain unchanged, and when implanted in vivo, we believe that degradation will occur over long time scales (months to years) and functionality will be maintained while the material remains mechanically stable, as previously observed in a 28 day in vivo study.^17^ Mesogen hydrophobicity coupled with intramesogen interactions also resulted in the accumulation of mesogen enriched degradation products in the material, which led to an increase in the nematic-to-isotropic transition temperature in LCEs. We observed this shift in both the accelerated and 150 day PBS control samples, suggesting that this effect was not only a result of degradation in an accelerated environment but could likely occur under biological conditions. This thermal effect may need to be considered in biological applications where the specific temperature associated with this transition is vital to the successful use of the LCE.

## Supplementary Material



## Notes

Comparison of 3D-printable chemistries formulation, quantitative values associated with DSC transitions, DSC traces for 3D-printable oligomers, degradation of 3D-printable chemistries in 1 M NaOH, water content comparison for BMEE:PEG formulations, image of fragmented sample, image of representative dried samples, polydomain mass loss and sample lengthening, thermal properties of PBS control samples, representative tensile tests for day 0 samples, all parallel tensile tests, all perpendicular tensile tests, representative dry WAXS/SAXS scattering patterns, representative hydrated WAXS/SAXS scattering patterns, WAXS/SAXS of PBS control samples, removal of degradation products in PBS samples, GPC data, ^1^H NMR spectra (PDF)
