# Supplementary material for: Engineering and Exploring Hydrolytic Degradation in 3D-Printed Liquid Crystalline Elastomers
Source: Biomacromolecules. 2026 Apr 3;27(5):3210–21. doi: 10.1021/acs.biomac.5c02638 (PMC13169365; doi:10.1021/acs.biomac.5c02638)
Supplement: Supplementary file 1 [file bm5c02638_si_001.pdf]

Supporting Information

Engineering and Exploring Hydrolytic Degradation in 3D-Printed Liquid Crystalline Elastomers

Lorin C. Danielsen[1,2], *Jason A. Burdick*[1,2*], *Timothy J. White*[1,3*]

[1]Department of Chemical and Biological Engineering, University of Colorado Boulder, Boulder, CO, 80303, USA

[2]BioFrontiers Institute, University of Colorado Boulder, Boulder, CO, 80303, USA

[3]Materials Science and Engineering Program, University of Colorado Boulder, Boulder, CO, 80303, USA

Table S1: Comparison of 3D-printable chemistries formulations

Table S2: Quantitative values associated with DSC transitions

Figure S1: DSC traces for 3D-printable oligomers

Figure S2: Degradation of 3D-printable chemistries in 1 M NaOH

Figure S3: Water content comparison for BMEE:PEG formulations

Figure S4: Degraded sample fragments

Figure S5: Representative dried samples

Figure S6: Polydomain mass loss and sample lengthening

Figure S7: Thermal properties of PBS control samples

Figure S8: Representative tensile tests for day 0 samples

Figure S9: All parallel tensile tests

Figure S10: All perpendicular tensile tests

Figure S11: Representative dry WAXS/SAXS scattering patterns

Figure S12: Representative hydrated WAXS/SAXS scattering patterns

Figure S13: WAXS/SAXS of PBS control samples

Figure S14: Removal of degradation products in PBS samples

Figure S15: GPC data

Figure S16: ^{1}H NMR spectra

Table S1. Comparison of 3D-printable chemistries used in this work. Theoretical molecular weights between crosslinks were calculated using the Carothers equation.

Chemistry	Liquid crystal mesogen (molecular weight)	Chain extender (molecular weight)	Crosslinker (molecular weight)	Ratio of monomers	Theoretical molecular weight between crosslinks (assuming 100% conversion)
Aza-Michael	C6M (672.76 g/mol)	DMHDAM (144.26 g/mol)	N/A	1:0.8	3940.84 g/mol
Thiol-Michael	C6M (672.76 g/mol)	HDT (150.31 g/mol)	N/A	1:0.8	3965.04 g/mol
Thiol-Michael/ thiol-ene	C6M (672.76 g/mol)	HDT (150.31 g/mol)	GBDA (254.32 g/mol) (~¼ of molecule caps each oligomer chain)	0.8:1:0.2	3506.17 g/mol

Table S2. Quantitative values associated with degraded sample DSC transitions in Figure 4.

	Transition temperature (ºC)	Area under transition peaks ((W/g) * ºC)
Day 0	49.20	0.26
Day 2	64.27	0.18
Day 4	68.11	0.23
Day 6	73.69	0.36
Day 8	90.56	0.83

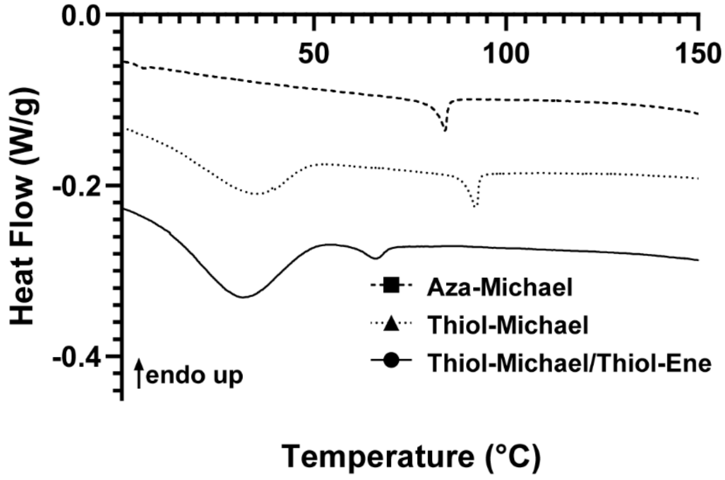

Figure S1. DSC traces for 3D-printable oligomers during cooling.

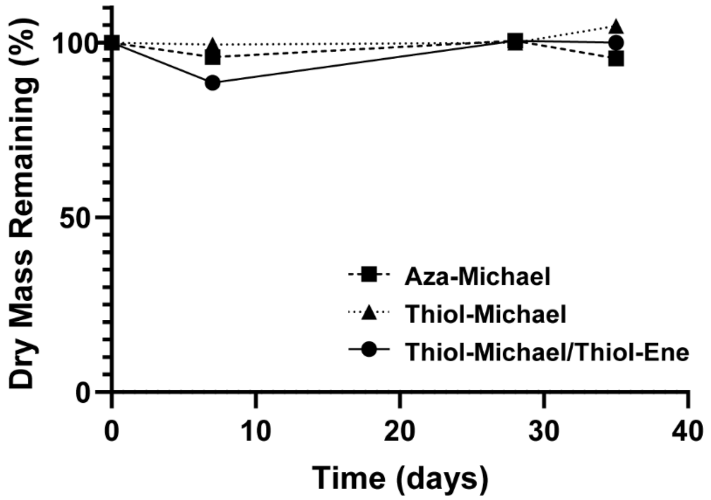

Figure S2. Preliminary mass loss study of 3D-printable chemistries degraded in 1 M NaOH. Since this was a preliminary study, n = 1.

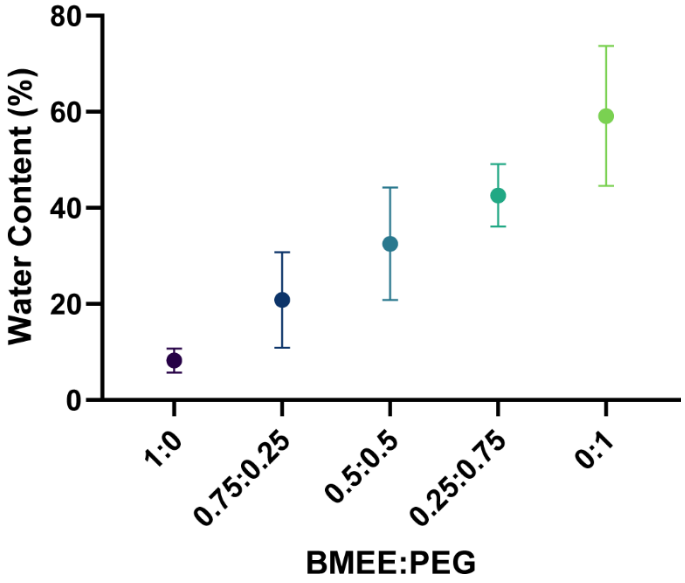

Figure S3. Weight content of hydrated BMEE:PEG samples after being soaked in PBS overnight. Data are reported as mean ± SD; n = 3.

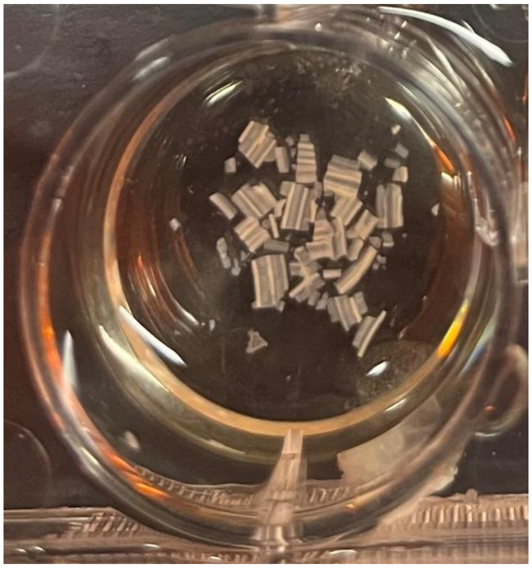

Figure S4. Day 10 degraded sample fragments in 1 M NaOH.

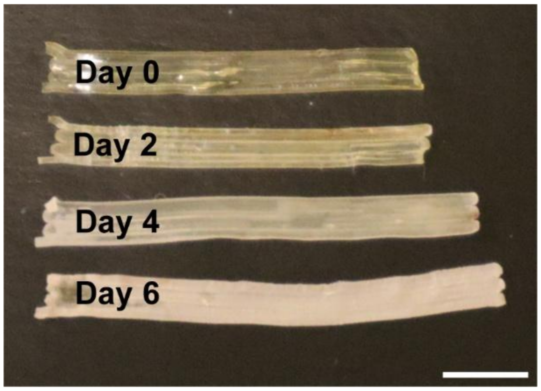

Figure S5. Representative dry samples for each time point, scale bar = 5mm.

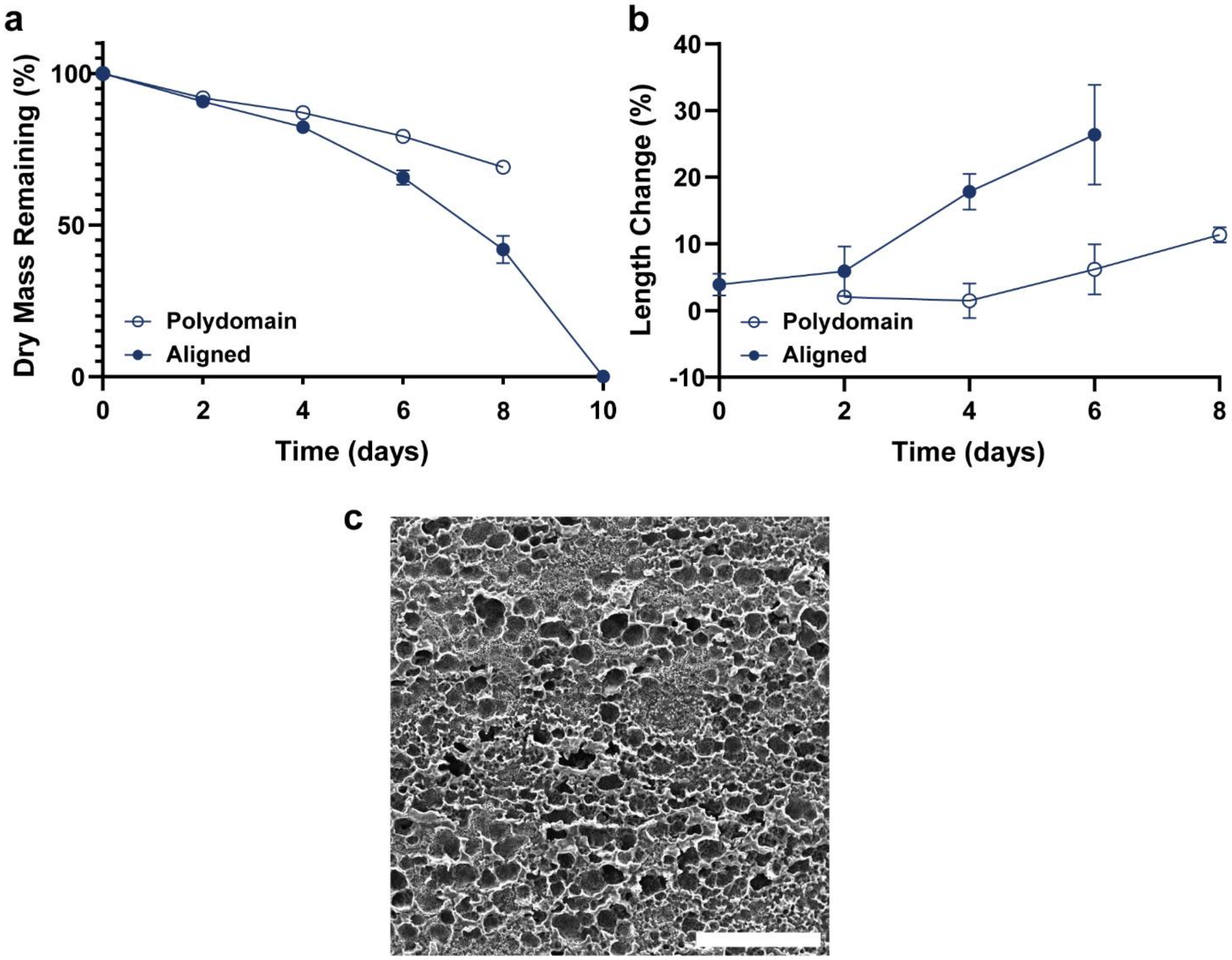

Figure S6. Polydomain versus aligned samples degraded in 1 M NaOH. (a) Mass loss; (b) length change; (c) SEM image of dried day 8 polydomain sample, scale bar = 250 µm. Data are reported as mean ± SD; n = 3. The difference in degradation rate is likely due to the sample heating used to eradicate mesogen alignment, which eliminates the surface ridges generated during 3D-printing and reduces the effective surface area.

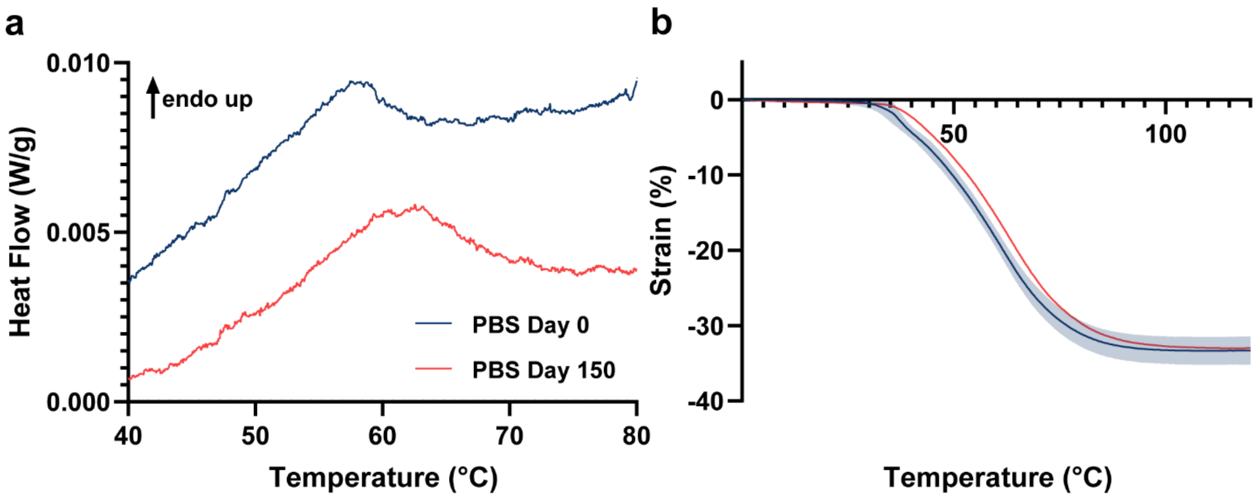

Figure S7. Day 0 and 150 PBS control samples. (a) DSC peaks corresponding to T_{ni}; (b) isostress tests. Data are reported as mean ± SD; n = 5 for PBS Day 0 isostress; n = 1 for all other data points.

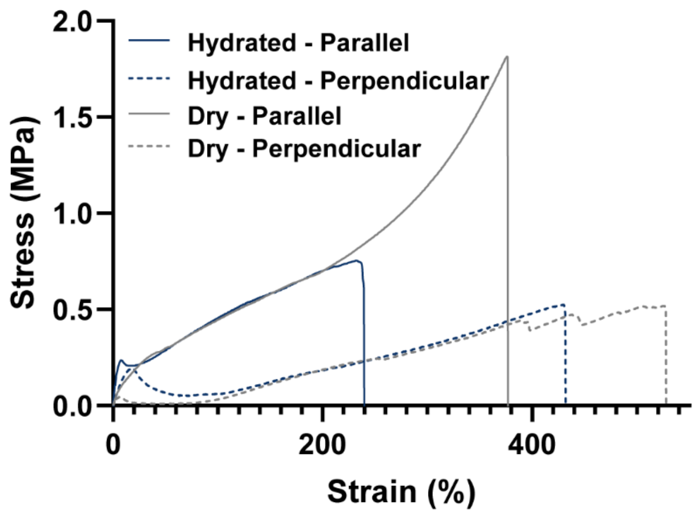

Figure S8. Representative tensile tests for day 0 hydrated and dry samples.

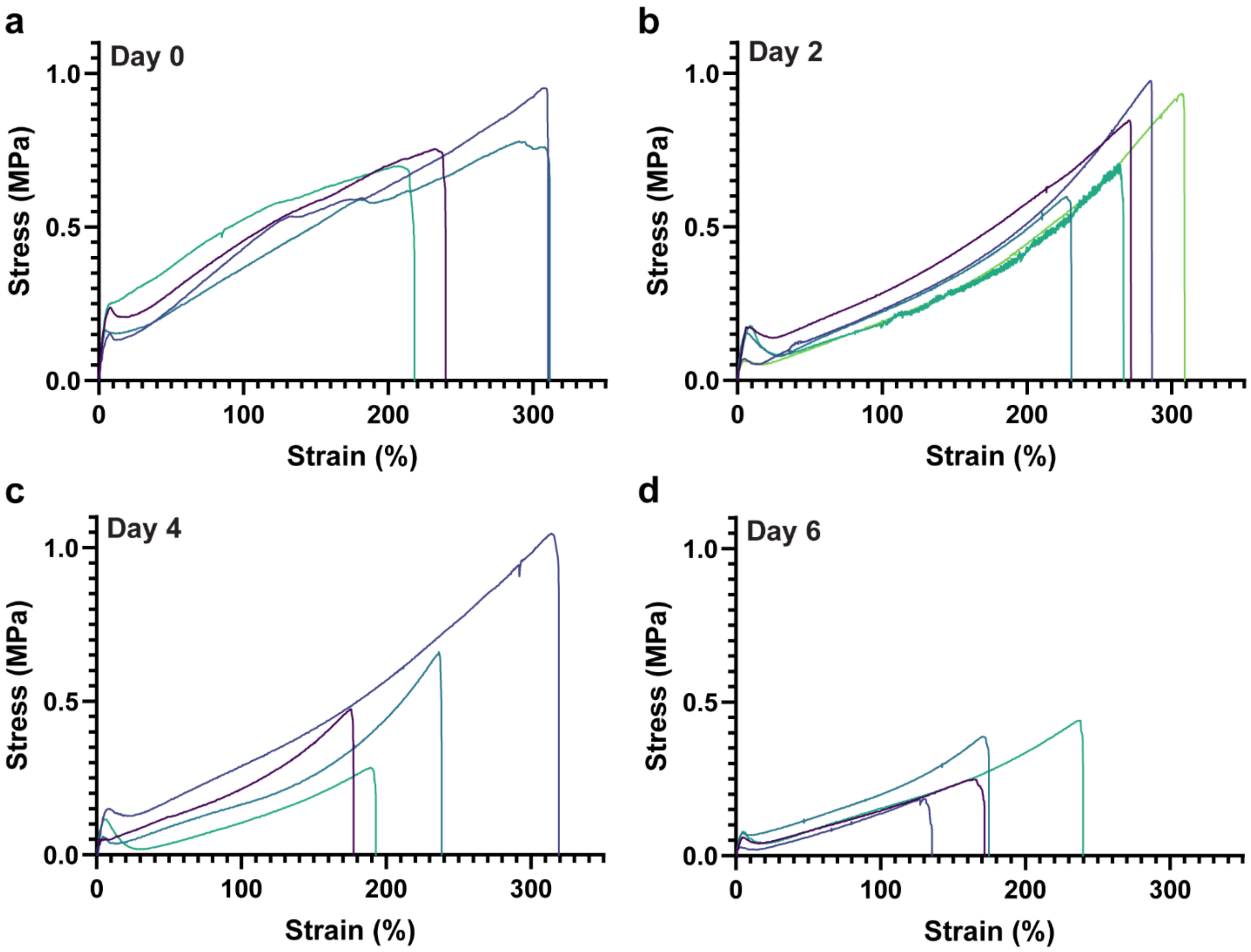

Figure S9. All parallel tensile tests. (a) Day 0; (b) day 2; (c) day 4; (d) day 6.

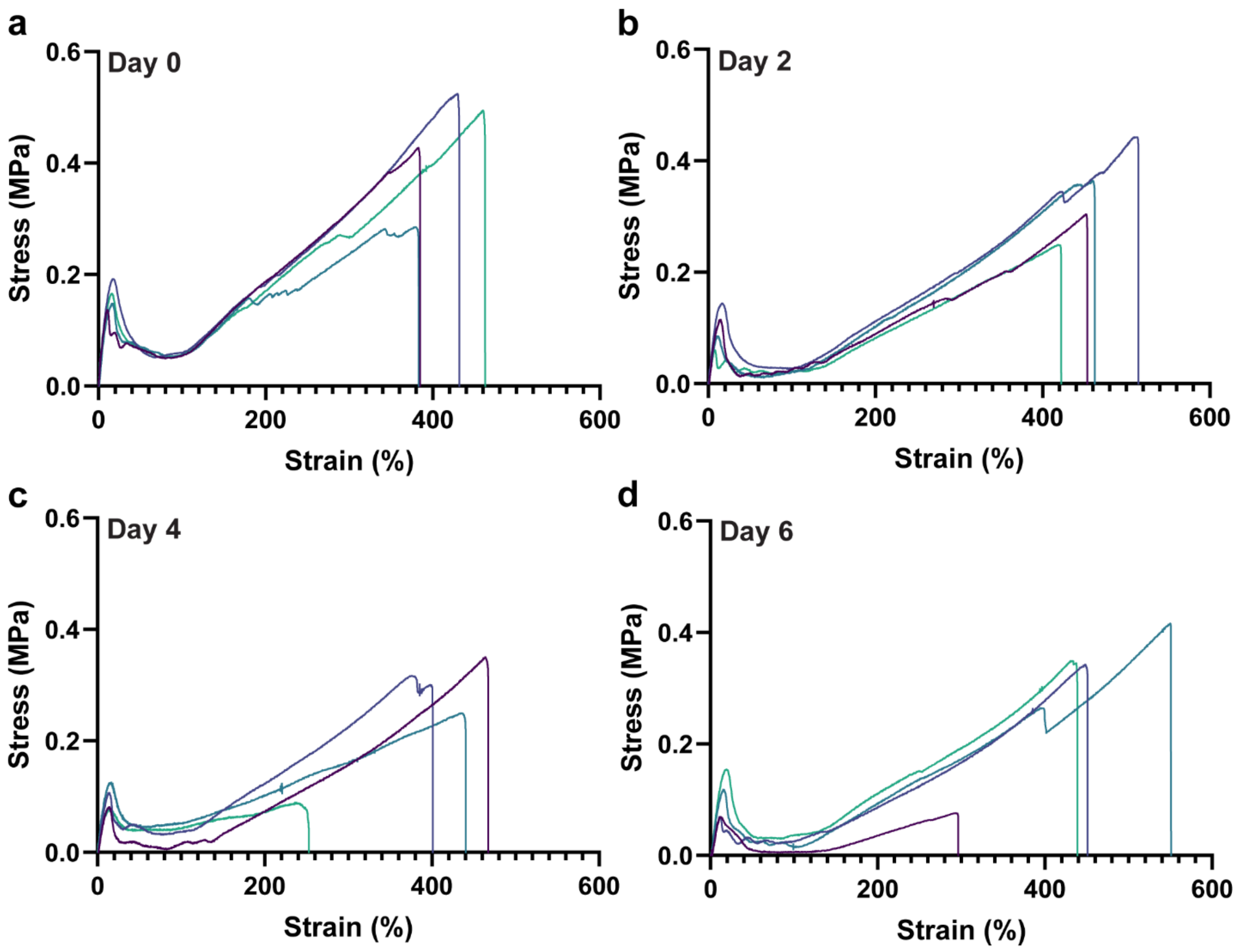

Figure S10. All perpendicular tensile tests. (a) Day 0; (b) day 2; (c) day 4; (d) day 6.

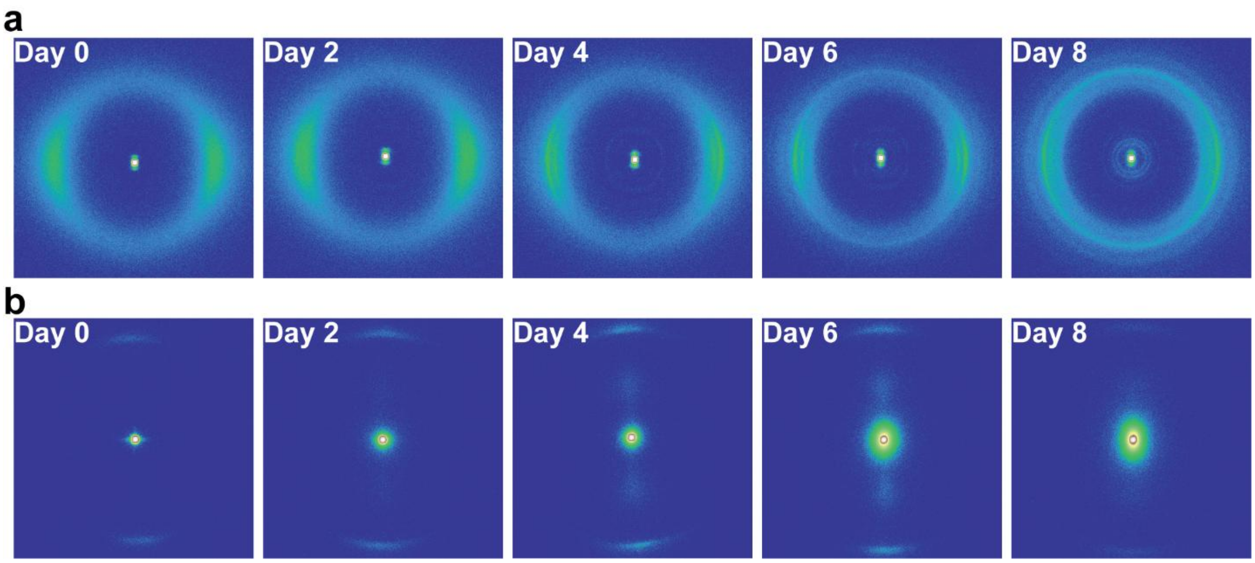

Figure S11. Representative (a) WAXS and (b) SAXS scattering patterns for dry samples.

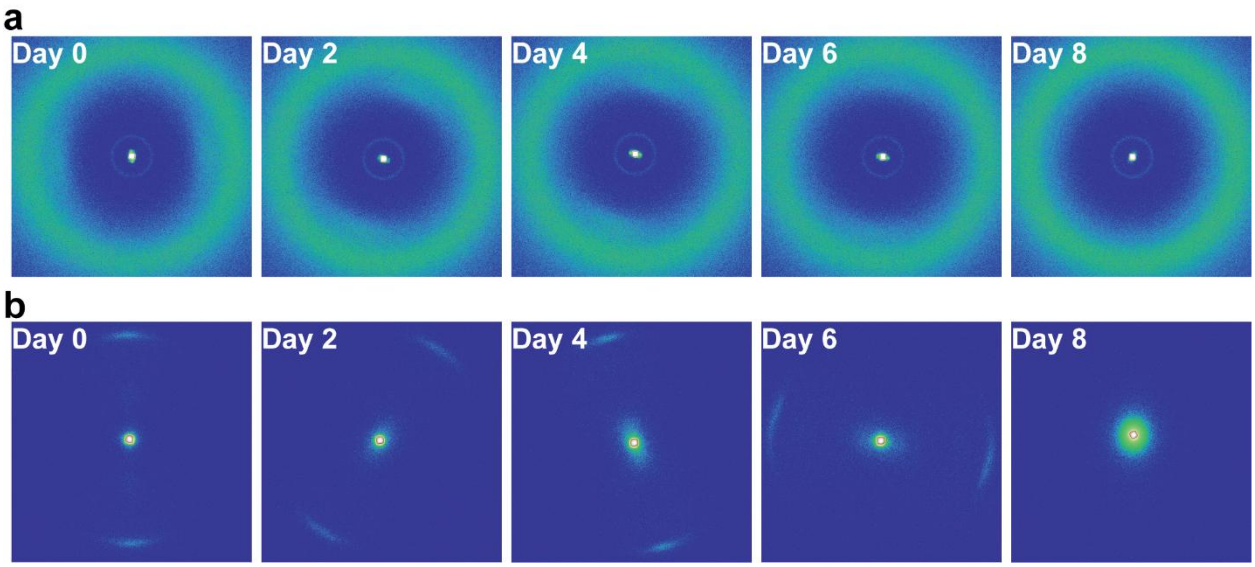

Figure S12. Representative (a) WAXS and (b) SAXS scattering patterns for hydrated samples.

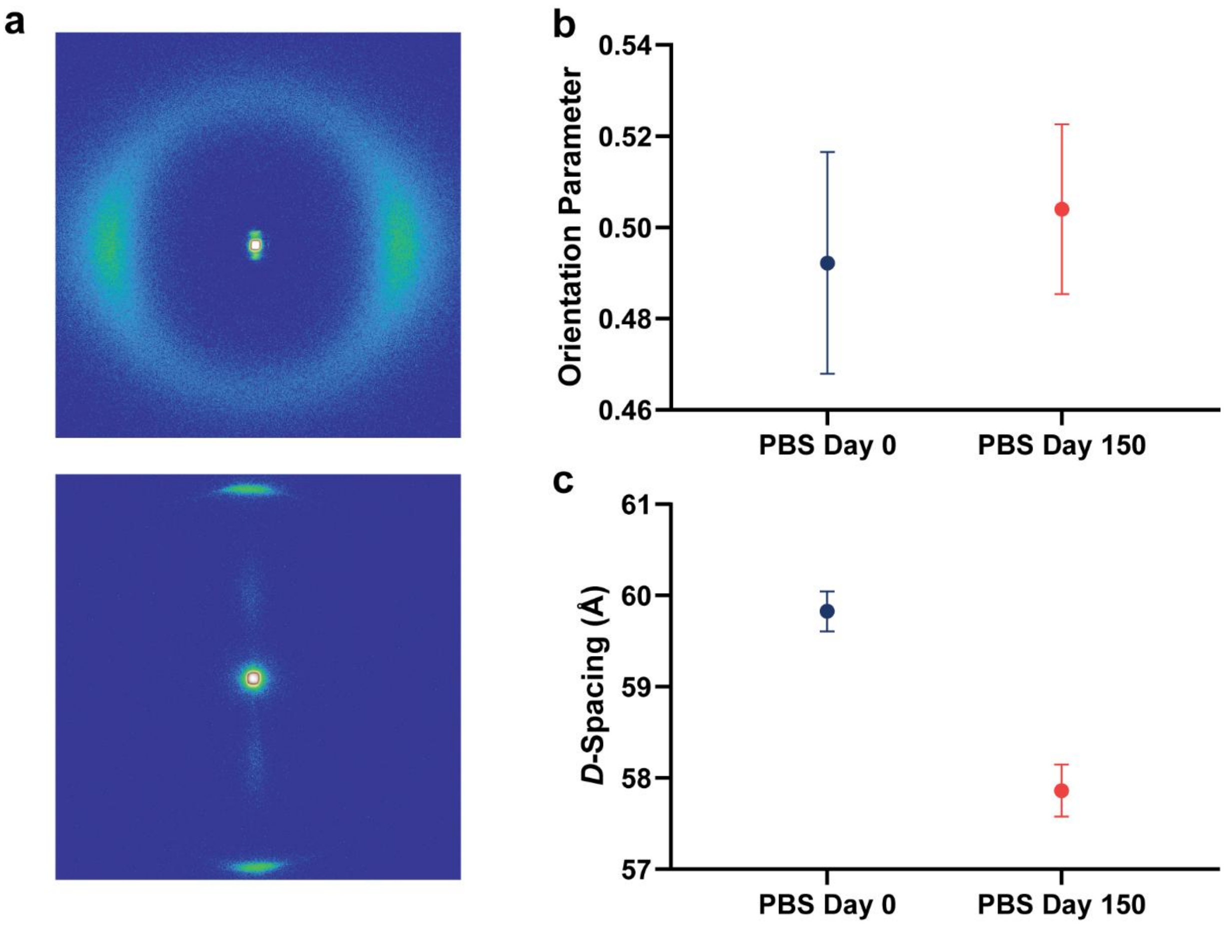

Figure S13. 150-day PBS control samples. (a) Representative WAXS and SAXS dry scattering patterns; (b) orientation parameters; (c) SAXS d-spacing. Data are reported as mean ± SD; n ≥ 3.

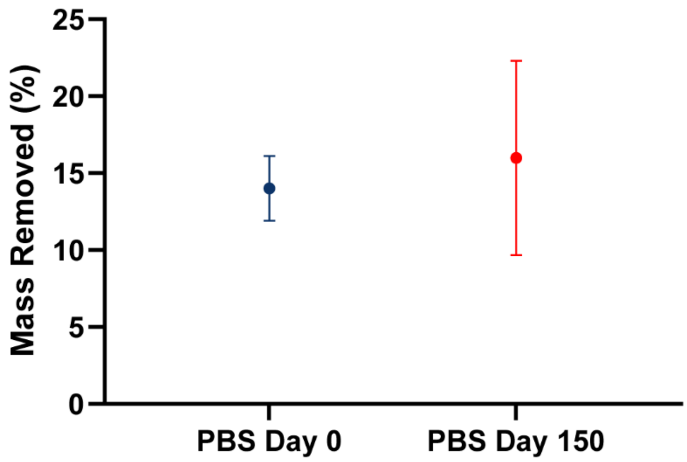

Figure S14. Removal of degradation products with acetone from PBS control samples. Data are reported as mean ± SD; n ≥ 3.

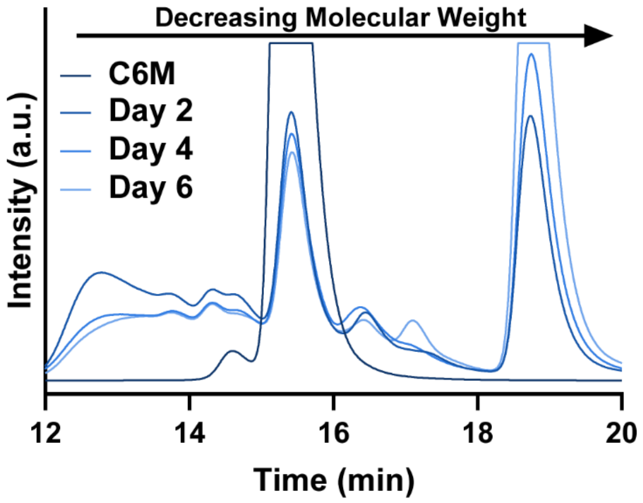

Figure S15. GPC data for just C6M and for retained degradation products at each degradation timepoint. UV absorption intensities are dependent on molar absorptivity of the compounds, so peak height does not necessarily correlate to concentration.

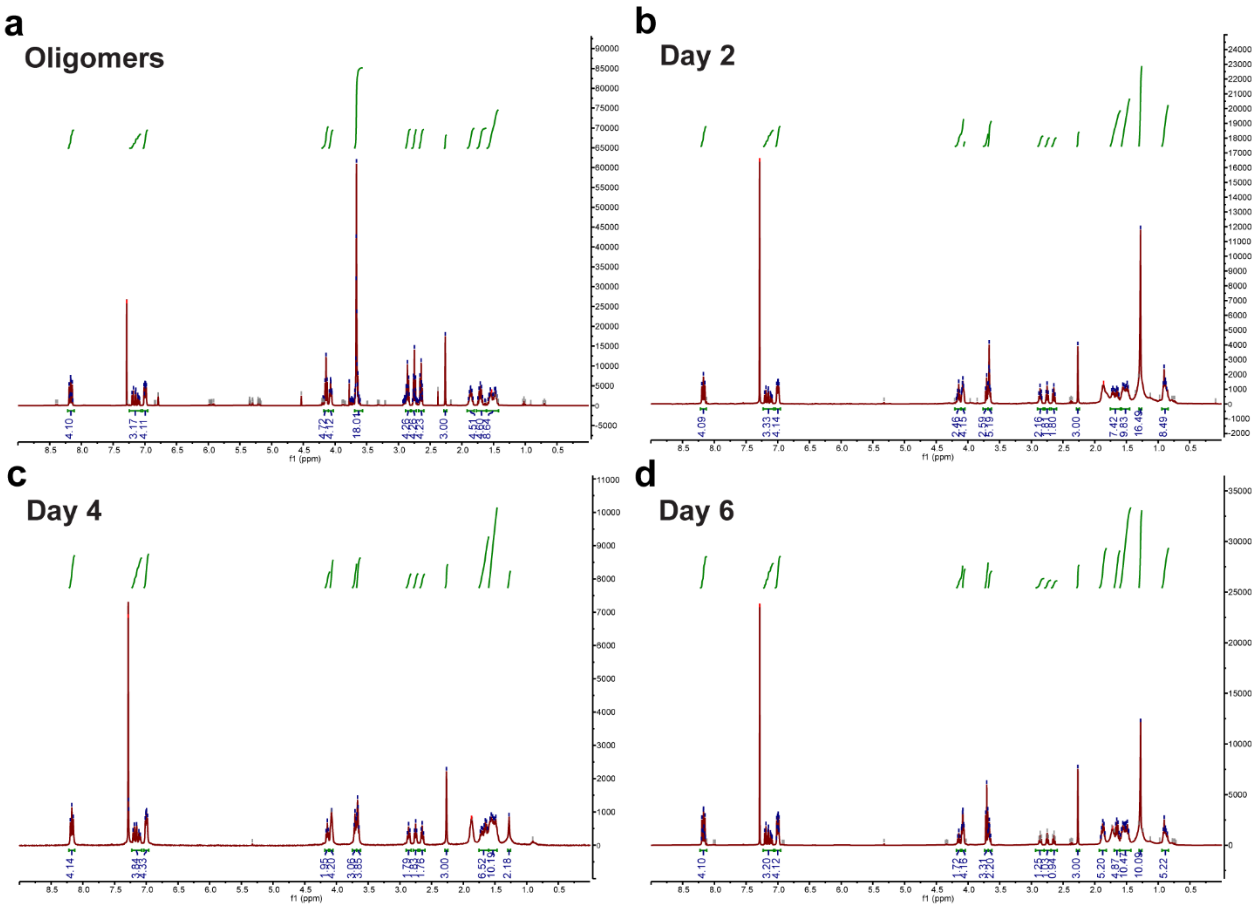

Figure S16. ^{1}H NMR spectra from Mestrenova with area under the peak values. (a) Oligomers; (b) day 2 retained degradation products; (c) day 4 retained degradation products; (d) day 6 retained degradation products.
